# Sensor Systems for Vehicle Environment Perception in a Highway Intelligent Space System

**DOI:** 10.3390/s140508513

**Published:** 2014-05-15

**Authors:** Xiaofeng Tang, Feng Gao, Guoyan Xu, Nenggen Ding, Yao Cai, Mingming Ma, Jianxing Liu

**Affiliations:** School of Transportation Science and Engineering, Beihang University, No.37 Xueyuan Road, Haidian District, Beijing 100191, China; E-Mails: tangjianhong@dae.buaa.edu.cn (X.T.); xuguoyan@buaa.edu.cn (G.X.); dingng@buaa.edu.cn (N.D.); yao.cai.east@gmail.com (Y.C.); malern@sina.com (M.M.); ljx@ae.buaa.edu.cn (J.L.)

**Keywords:** Highway Intelligent Space System, space sensors, vehicle-mounted sensors system, model predictive control, vehicle environment perception

## Abstract

A Highway Intelligent Space System (HISS) is proposed to study vehicle environment perception in this paper. The nature of HISS is that a space sensors system using laser, ultrasonic or radar sensors are installed in a highway environment and communication technology is used to realize the information exchange between the HISS server and vehicles, which provides vehicles with the surrounding road information. Considering the high-speed feature of vehicles on highways, when vehicles will be passing a road ahead that is prone to accidents, the vehicle driving state should be predicted to ensure drivers have road environment perception information in advance, thereby ensuring vehicle driving safety and stability. In order to verify the accuracy and feasibility of the HISS, a traditional vehicle-mounted sensor system for environment perception is used to obtain the relative driving state. Furthermore, an inter-vehicle dynamics model is built and model predictive control approach is used to predict the driving state in the following period. Finally, the simulation results shows that using the HISS for environment perception can arrive at the same results detected by a traditional vehicle-mounted sensors system. Meanwhile, we can further draw the conclusion that using HISS to realize vehicle environment perception can ensure system stability, thereby demonstrating the method's feasibility.

## 1. Introduction

Vehicle-highway information systems have been a hotspot in intelligent transportation system research. As an important part in intelligent transportation systems, intelligent vehicles mainly use vehicle-mounted sensor systems, information processing systems and control systems, *etc.* to perceive the surrounding environment. Vehicle-mounted sensor systems used in intelligent vehicles provide a greatly improved level of vehicle environment identification. However, due to the increased complexity of the highway environment, the limited range of vehicle environment perception, especially the shortcoming that multiple vehicle-mounted sensor systems traveling in the same direction produce interference, traditional vehicle perception systems cannot produce comprehensive environment perception. Therefore, a Highway Intelligent Space System (HISS) is proposed to solve these problems. Using the HISS to realize vehicle environment perception is expected to be the next trend in the intelligent vehicle field in the future.

The traditional environment perception environment uses sensors such as laser-based sensors, millimeter-wave radars and vision-based sensors, *etc.* installed on vehicles. The Toyota system uses millimeter-wave radars and CCD sensors to detect the distance between the objective vehicle and the target vehicle. Carnegie Mellon University researched Navlab that uses trinocular vision and range finder to increase vehicle safety features and a GPS receiver is used to detect vehicle positioning and laser-based sensors are used to detect obstacles to realize active vehicle safety [1]. A commercially automated vehicle has been designed by the Federal University of Espirito Santo, which has been actively working on obstacle detection using stereo vision [2]. The key research content concerning intelligent vehicles includes lane departure warning, collision warning, advanced driver assistance systems and automated platooned driving, *etc.* The most important task of intelligent vehicles is localization of the vehicle and detection of other vehicles as well. However, the detection of other vehicles can use a variety of visual sensory systems, or laser to ultrasonic sensors. The visual system can fail under low visibility conditions, in low illumination environments or in heavy traffic [3]. The principle of laser sensors is generally robust, but they have reduced sensitivity in bad weather conditions [3]. Radars are cheap, but may fail in the presence of nearby reflective objects. Ultrasonic sensors can be used only at short distances and due to their measurement characteristics, they may fail in some cases [3]. Recently, scholars have proposed the new idea of putting sensors and control systems in the highway environment to realize information exchange between the infrastructure and vehicles. Generally the fundamental communication technology based on FoF communication technology [4], analysis model for defining transmission speed [5], multi-agent information transmission methods [6], *etc.* is used to achieve the information exchange process. Seoul National University describes the development of an infrastructure-based path-tracking control system. The control system consists of an infrastructure sensor module, a vehicle controller and actuator modules. Some relative parameters such as vehicle position, heading angle and obstacles surrounding the vehicle from an infrastructure sensor module, *etc.* will be sent to vehicles [7]. The innovative method realizes information exchange mainly by the vehicle and infrastructure sensor module.

In this paper, we have proposed the HISS to realize vehicle environment perception. The HISS consists of infrastructure sensor modules, system servers and communication devices, *etc.* The most notable features of the HISS show that the information obtained from infrastructure sensors is first sent to system servers, the effective information will then be sent to vehicles by servers according to the specific driving situation. Secondly, the HISS can be used to perceive some sensitive vehicles on the road ahead and make vehicles identify the surrounding environment in advance, which enhances vehicle safety driving characteristics. The main research topic in this paper is to evaluate the function of HISS compared with traditional vehicle-mounted sensor systems. Considering the faster driving characteristics of vehicles on highways, some effective information should be predicted in advance to make vehicles follow the specified route to avoid the accidents. Therefore, we research the driving situation results to evaluate the program. First, the vehicle driving states such as velocity, distance, *etc.* in the HISS environment are obtained from space sensors. Second, the vehicle driving states in traditional vehicle-mounted sensors system are gathered. Third, the inter-vehicle kinematics are built and a model predictive control approach is proposed to estimate vehicle driving states in the next time. Meanwhile, the actual vehicle driving states obtained from the above two approaches are regarded as the reference inputs for a Model Predictive Control (MPC) approach. Furthermore, through analysis of the errors between the estimated driving states and the actual vehicle driving states, we can draw the conclusion that HISS is used to realize vehicle environment perception, which can improve vehicle environment perception ability.

The remainder of this paper is organized as follows: Section 2 addresses the basic principles of HISS and the vehicle driving states under the HISS environment. Section 3 shows the vehicle driving states detected by vehicle-mounted sensors system. Section 4 presents the inter-vehicle dynamics model and model predictive control theory. Finally, comparative analysis of the experimental results is summarized.

## 2. HISS Principle

### 2.1. The Basic Theory

**Figure 1.**
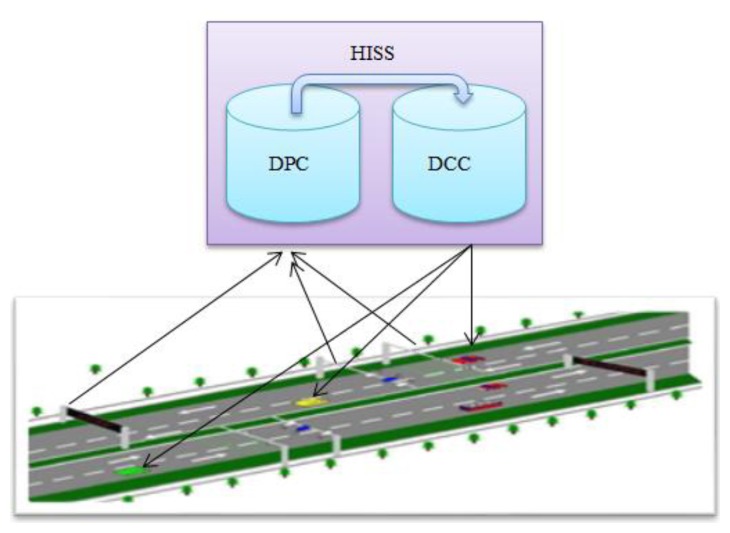
HISS principle.

HISS is composed of the highway environment and vehicles. The basic principle is that distributed sensors are installed in the enclosed highway environment and communication technology is used to realize the information exchange between individual vehicles and information servers, thereby the realization of context-ware information to be shared throughout the overall space. The basic principle is shown in Figure 1.

From Figure 1, we can show that HISS mainly includes three parts: sensor devices, communication devices and servers. Sensor and communication devices mean that various sensors such as visual sensory systems, laser to ultrasonic or radar, *etc.* and wireless networks, mobile network stations and computing devices. Servers are included in the data processing center (DPC) and the data communication center (DCC). DPC is responsible for processing the sensor data collected from the space sensors, including information integration, data processing, information prediction and mathematical models analysis; the DCC is responsible for sending the new processed data from the DPC. The effective information is sent to vehicles.

**Figure 2.**
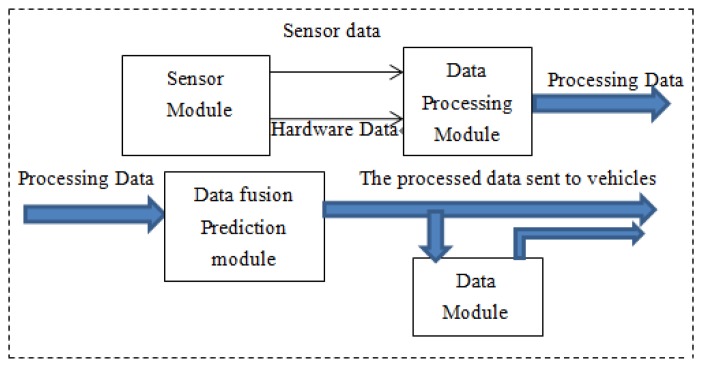
Information process module.

The HISS' embedded communication and multi-model sensing devices have the function of environmental information perception, computing process and information exchange and dissemination. When a vehicle is passing some highway sections that are prone to accidents, the effective information is acquired from the space sensors, and after processing by servers, the effective information packets are sent to the specific vehicles. Figure 2 depicts the processing of sensor data.

**Figure 3.**
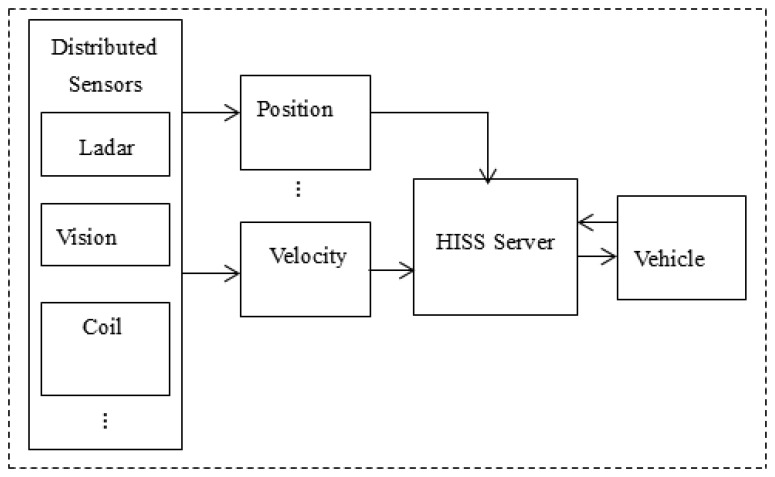
The hardware sensor structure.

Sensor modules are responsible for collecting and outputting the raw data, and provide the hardware information to the HISS servers to facilitate their own identification. The data processing module mainly receives the raw data. The raw data is analyzed and synthesized to process the desired information. The information fusion prediction module will analyze the data sent from the data processing module, and after a multi-sensor fusion prediction process, the final effective data is sent to vehicles. HISS has a large number of distributed visual sensory systems, or laser to ultrasonic, *etc.* which form a large amount of vehicle driving state detection and sensor information fusion data. The system improves the road recognition ability and provides more comprehensive information for safe vehicle driving. Figure 3 shows the hardware sensor structure.

### 2.2. Vehicle Driving State Acquisition under HISS Environment

**Figure 4.**
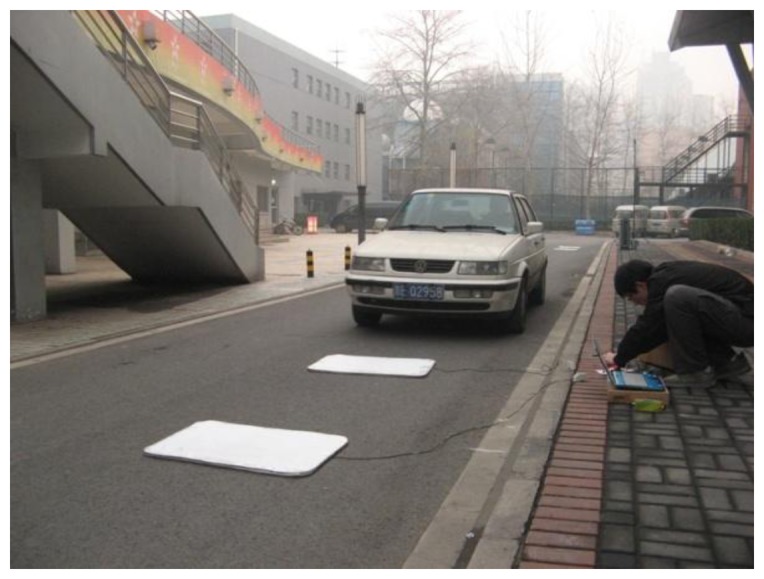
The test process.

In order to the research vehicle environment perception problem under a HISS environment, an initial HISS experimental platform was built to study sensor perception, vehicle detection and information fusion. The main experimental devices are as follows: two experimental vehicles, a self-developed test machine for intelligent vehicles, sensors (two sets of laser sensors, one millimeter-wave radar, one binocular stereo vision), one intelligent vehicle simulation platform, one WiFi experimental system and a video capture system, *etc.* All the equipment can support the HISS plan, vehicle driving state acquisition and information fusion, *etc.* of simulation tests. For the project, a safe campus highway about 200 m in length is chosen as the experimental section. Machine vision cameras and magnetic induction coil detectors are used for acquiring vehicle driving states. The sensors are installed as follows: two CCD cameras are placed at intervals of 70 m and magnetic induction coil detectors are installed between the CCD camera fields of vision. The schematics of the processes are shown in [8,9] and Figure 4.

References [8,9] and Figure 4 indicate the vehicle distance collection approach: a tripod is used to install the cameras. In addition, the information exchange between the computer and cameras is achieved by a USB interface. The pinhole model distance principle is used for distance measurement. For this experiment, vehicle speed will remain about 20–45 km/h. According to the vehicle speed range, the vehicle driving distance range is calculated to be about (5.6 m, 12.5 m), while the frequency of video camera is about 31 frame/s. Therefore, the range of distances for each frame field of vision lies in the (17.9 cm, 40.3 cm) range. When the vehicle enters the range of the second set of cameras, the distance between the vehicle and the first camera is about 45.6 m. Considering the characteristics of real-time information, the vehicle distance measured from the first set of cameras is greater than 45.6 cm in the first frame. According to the experimental results [9], the first vehicle speed is chosen as *v*_1_ = 10.64 m/s, the second vehicle velocity is *v*_2_ = 11.10 m/s. At this moment, the distance between vehicle 1 and vehicle 2 is 16.67 m as an analysis basis.

## 3. Vehicle Driving State Acquisition under Vehicle-Mounted Sensors System Environment

**Figure 5.**
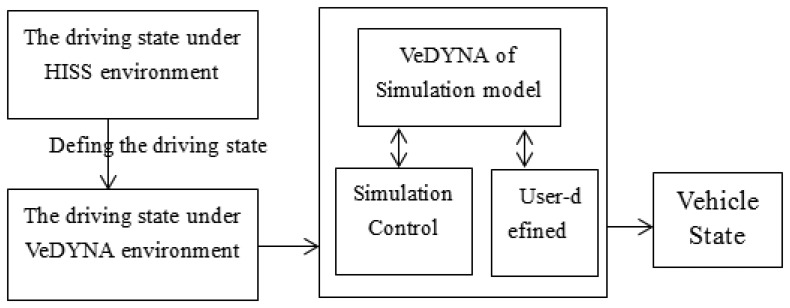
Vehicle driving state output under the veDYNA environment.

The traditional vehicle-mounted sensors system for vehicle driving state acquisition is achieved in the veDYNA software environment, which provides scholars with real-time virtual vehicle ability. The veDYNA Traffic Add-on is a versatile simulation tool for the development and testing of vehicle control systems in a traffic environment. The traffic environment can be defined by up to 16 fellow cars and up to 64 stationary obstacles. Especially driver assistance systems such as ACC or lane departure warning systems that pick up environmental information by means of radar, lidar or ultrasonic sensors can be tested in a time-efficient way under well-defined and reproducible conditions in the laboratory without danger for drivers or prototypes [10]. The process for using traditional vehicle-mounted sensors system for obtaining vehicle driving state is that first, some relative parameters are set that are equal to the parameters for HISS to solve the relative driving state parameters such as vehicle distance, relative speed, *etc.* These parameters are regarded as the reference parameters of the traditional vehicle-mounted sensor system. The basic principle of the system is shown in Figure 5.

**Figure 6.**
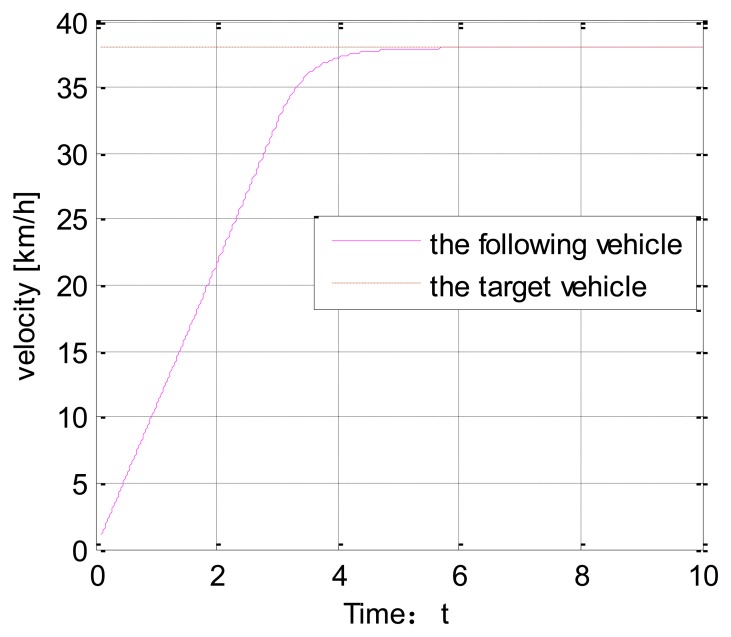
Vehicle speed change.

**Figure 7.**
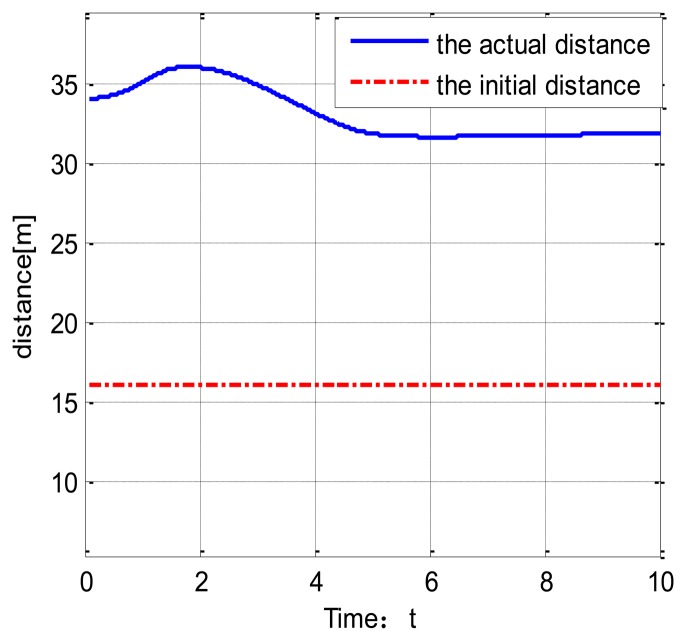
The distance between the two vehicles.

Second, the manipulation mode, road environment, driver parameters and simulation parameters in the veDYNA environment are set to the suitable state. The simulation results of the traditional vehicle state are as follows:

Figure 6 shows the vehicle speed change under the veDYNA environment. Suppose the target vehicle drives at a speed of 37 km/h. The following vehicle tracks the target vehicle at t = 4 s. Figure 7 shows the distance between the two vehicles. The red line stands for the initial distance. The blue line stands for the actual distance. From Figure 7, we can show that the actual distance between the two vehicles is 16 m.

## 4. System Model

**Figure 8.**
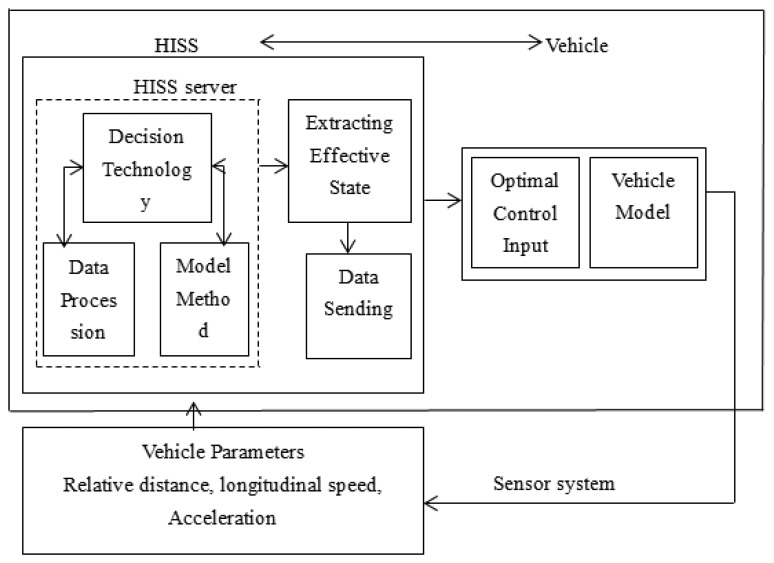
The specific theory analysis method.

The effect of the HISS is to provide the vehicle with information about the road in front of it. Therefore, making full use of HISS servers for vehicle dynamics model considers the highway situation in front of the vehicles. When the vehicle drives at a high speed, the road ahead should be obtained in advance to help drivers adjust to its driving state. The specific theory analysis method is shown Figure 8.

Figure 8 shows the procedure whereby space sensors are used to obtain vehicle driving state such as vehicle velocity, acceleration, relative velocity, relative distance, *etc.* and are sent to the HISS server. Meanwhile, the processed data is regarded as the vehicle driving state at time t. Second, vehicle dynamics model is built and model predictive control approach is used to predict vehicle driving state at the next timepoint. During the prediction process, vehicle driving state data stored in the HISS server are regarded as the reference value, so that by solving the optimization function obtains the optimal sequence of future control input value. Meanwhile, the effective information should be extracted from the HISS server and is sent to vehicles so drivers can adjust in real-time their driving situation, thereby tracking the predicted driving state to ensure vehicle environment perception in advance.

A model predictive control approach (MPC) is used to predict the vehicle driving state. It is a natural control framework to deal with the design of coordinated, distributed control systems because of its ability to handle input and state constraints, and also because it can account for the actions of other actuators in computing the control action of a given set of control actuators in real-time [11]. MPC mainly includes three parts: prediction model, rolling optimization and feedback correction.

### 4.1. Vehicle Dynamics Model

**Figure 9.**
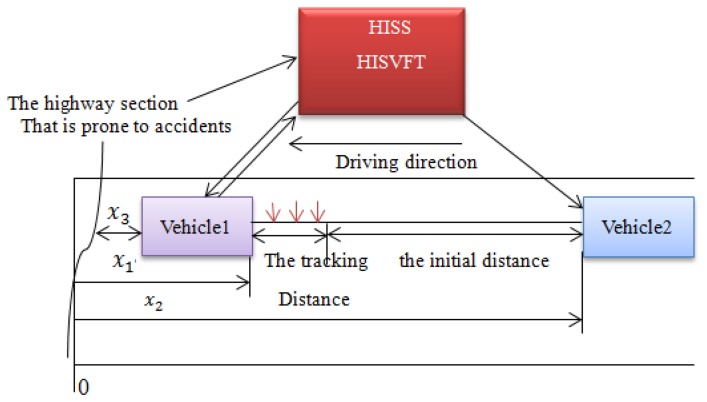
The vehicle dynamics model.

Suppose that the two vehicles are driving towards some highway sections that are prone to traffic accidents. The vehicle dynamics model considering the HISS servers is built in Figure 9.

As can be seen from Figure 9, suppose that an accident has happened about *x*_3_ m ahead of vehicle 1. With the HISS server, the road information ahead will be sent to vehicle 1 and vehicle 2. Moreover, the initial distance between the two vehicles is long. At this moment, vehicle 1 will adjust its vehicle velocity instantly, and vehicle 2 can obtain vehicle 1's driving state from the HISS server. When vehicle 2 enters the tracking distance, model predictive control is used to predict the driving state. While the reference states are obtained by the HISS environment and traditional vehicle-mounted sensors system, respectively.

Because vehicle 1's speed and acceleration are uncontrollable, we should control vehicle 2 to ensure a suitable relative distance and relative speed to avoid accidents. The vehicle dynamics model and state constraints should be built as follows: for the purpose of analyzing the inter-vehicle driving situation, first, the inter-vehicle dynamics model is designed between the leading vehicle 1 and vehicle 2 as follows:

(1) $$\begin{array}{c} {\dot{v}}_{f}=a_{f}\\ \xi_{d}=l_{l}-l_{f}\\ \Delta v=v_{l}-v_{f}\\ \;\xi_{e}=\xi_{d}-d_{\text{des}} \end{array}$$

where, *ξ_d_* is the inter-vehicle distance, Δ*ν* is the error between vehicle 1 and vehicle 2, *ξ_e_* is the error between the actual inter-vehicle distance and the desired vehicle safety distance. *ν_l_* is vehicle 1's speed and *ν_f_* is vehicle 2's speed, respectively, and *a_f_* is the acceleration of vehicle 2.

Second, the longitudinal dynamics of vehicle 2 are nonlinear. According to the vehicle dynamics in [12], the longitudinal dynamics of vehicle 2 are transferred as follows:

(2) $$\begin{array}{c} {\dot{a}}_{f}=g_{f}\left(v_{f},a_{f}\right)+h_{f}(v_{f})\delta_{f}\\ g_{f}\left(v_{f},a_{f}\right)=-\frac{2K_{ad}}{m}-\frac{1}{\tau_{f}}[a_{f}+\frac{K_{ad}}{m_{f}}v_{f}^{2}+\frac{K_{md}}{m_{f}}]\\ h_{f}\left(v_{f}\right)=\frac{1}{m_{f}}\tau_{f} \end{array}$$

If the parameters in Equation (2) are exactly known, the following feedback linearizing control law could be adopted:

(3) $$\delta_{f}=m_{f}\mu_{fc}+K_{ad}v_{f}^{2}+K_{md}+2\tau_{f}K_{ad}v_{f}a_{f}$$

where, *μ_fc_* is the input signal that makes the closed loop system satisfy certain performance criteria. In the controller (3), we achieve the following objectives:

(1) The feedback linearization results in a linear system, as discussed above. However uncertainties in parameters can potentially make the linearization process inexact. The study of such a case would be an interesting topic to be considered in future research.
(2) The simplification of the system model by excluding some characteristic parameters (e.g., the mechanical drag, mass and air resistance) from the vehicle dynamics. Manipulating Equation (1) through Equation (3), the equation becomes:

(4) $${\dot{\alpha }}_{f}=\frac{1}{\tau_{f}}(\mu_{f_{c}}-\alpha_{f})$$

where, *τ_f_* is the engine time constant and its value is 0.25, a single parameter that describes the dynamics of the propulsion system and internal disturbances. *μ_fc_* can be viewed as the throttle/brake input causing acceleration/deceleration in the controlled vehicle. The system thus takes the form which can be described by the following standard equations:

(5) $$\left[\begin{array}{l} {\dot{v}}_{f}\\ {\dot{\xi }}_{e}\\ \overset{\cdot }{\Delta v}\\ {\dot{\alpha }}_{f} \end{array}\right]=\left[\begin{array}{cccc} 0 & 0 & 0 & 1\\ 0 & 0 & 1 & -h_{i}\\ 0 & 0 & 0 & -1\\ 0 & 0 & 0 & -1/\tau_{f} \end{array}\right]\cdot \left[\begin{array}{c} v_{f}\\ \xi_{e}\\ \Delta v\\ \alpha_{f} \end{array}\right]+\left[\begin{array}{c} 0\\ 0\\ 0\\ 1/\tau_{f} \end{array}\right]\cdot \left[u(t)\right]+\left[\begin{array}{c} 0\\ 0\\ 1\\ 0 \end{array}\right]\cdot \left[\alpha_{l}\right]$$

(6) $$\text{y}\left(\text{t}\right)=\left[\begin{array}{cccc} 1 & 0 & 0 & 0\\ 0 & 1 & 0 & 0\\ 0 & 0 & 1 & 0\\ 0 & 0 & 0 & 1 \end{array}\right]\cdot \left[\begin{array}{c} v_{f}\\ \xi_{e}\\ \Delta v\\ \alpha_{f} \end{array}\right]$$

Writing the above equation as standard state-space equations, we have:

(7) $$\dot{x}\left(t\right)=Ax\left(t\right)+Bu\left(t\right)+Cv(t)$$

(8) y(t)=Dx(t)

where, $A=\left[\begin{array}{cccc} 0 & 0 & 0 & 1\\ 0 & 0 & 1 & -h_{i}\\ 0 & 0 & 0 & -1\\ 0 & 0 & 0 & -1/\tau_{f} \end{array}\right],\;B=\left[\begin{array}{c} 0\\ 0\\ 0\\ 1/\tau_{f} \end{array}\right],\;C=\left[\begin{array}{cccc} 1 & 0 & 0 & 0\\ 0 & 1 & 0 & 0\\ 0 & 0 & 1 & 0\\ 0 & 0 & 0 & 1 \end{array}\right],D=\left[\begin{array}{c} 0\\ 0\\ 1\\ 0 \end{array}\right]$where, *ξ_e_* is the amount of error or drift from this desired spacing. State variables are x(t) = [*ν_f_ ξ_e_* Δ*ν α_f_*]*^T^* and *ẋ*_2_(*t*),*ẋ*_3_(*t*) represent relative velocity and acceleration of vehicle 2, respectively. *ν*(*t*) is vehicle 1's deceleration.

### 4.2. Controller Design and Optimal Sequence

We analyze the state relation between leading vehicle 1 and vehicle 2 to ensure vehicle platoon safety. Usually, the so-called safety distance is defined as this desired distance, yielding:

(9) $$x_{r,d}\left(k\right)=x_{r,0}+v_{h}(k)t_{hw,d}$$

with *x_r_*_,0_ is a constant representing the desired distance at standstill, and the desired headway time *t_hw_*_,_*_d_* is a measure of the time it takes to reach the current position of the preceding vehicle if the following vehicle continues to drive with its current velocity, *i.e.*, for constant *v_h_*(*k*). Correspondingly, the tracking error at discrete time *k* is defined as *ξ_e_*(*k*) = *x_r_*_,_*_d_*(*k*) − *x_r_*(*k*). In order to avoid a crash, we may control the distance error to approximate zero in steady tracking state or a positive value that the system is different from other traditional vehicle safety systems. Besides the above primary control targets, several secondary objectives, related to the key characteristics, in this case safety, have to be included. Regarding safety, besides control of the relative position, the relative speed should be kept small.

As to constraints of the relative distance error, when vehicle 1 runs uniformly, larger of smaller inter-vehicle distance may occur in real time. The inequality for sub-objective is *ξ_e_max_* where, *ξ_e_min_* = −5*m* is the lower boundary which is again obtained from the driver experimental data, and *ξ_e_max_* ≤ 6 *m* is the higher boundary in the literature [13]. The weight *Q_e_* which is the weight on the error *ξ_e_* between the desired and the actual distance has to be considered. The larger *Q_e_*, the smaller the time reaches a steady-state situation. Although the focus is on safety, it has to be remarked that for increasing *Q_e_*, the acceleration will increase as well [14]. Regarding the relative speed constraint, we assume that when vehicles arrive at a tunnel ahead, vehicle velocity is 0 ≤ v_f_ ≤ 80 km/h. The relative velocity should be minimized as fast as possible. We research the range in −1 ≤ Δv ≤ 0.9 m/s We define a constant weight value *Q_νr_* to ensure an average desirable behavior. The vehicle 2's acceleration constraint is defined in the range *a_f_*___*_min_* = −3.0ms^−2^, *a_h_*_,_*_max_* ∈ [2.0,3.0]ms^−2^. The absolute value of *α_f_*___*_min_* being bigger than that of *α_f_*___*_max_* can accommodate a larger braking degree to prevent rear-end collisions [14]. The control input constraint includes throttle input or brake input, so we could define the control formulation as *u*(*k*) ∈ [−1 1].

Based on the above research content, we propose a model predictive control approach to solve the MPC problem with constraints for vehicle 1. Considering the fact that an MPC approach is usually designed and implemented in the discrete-time domain, the continuous-time (7) is converted into a discrete-time model.

The cost criterion is typically formulated as a linear or a quadratic criterion. As MPC is used, a cost criterion *J*, which is minimized over a prediction horizon *N_p_*, has to be defined. The prediction is performed within an optimization window *N_p_*, which is the number of samples and each sample is denoted by the time *k_i_*. At each sampling instant *k_i_*, the state information vector *x*(*k_i_*) is measured, which provides the current plant information. Having the current plant information *x*(*k_i_*), the future system states are predicted for *N_p_* number of samples, the future state variables can be defined as follows:

$$x(k_{i}+1)/k_{i},x(k_{i}+2)/k_{i},\cdots ,x\left(k_{i}+m\right)/k_{i},\cdots ,x\left(k_{i}+N_{p}\right)/k_{i}:=x\left(k_{i}+m\right)/k_{i},m=0,1,\cdots ,N_{p}$$

where, *x*(*k_i_* + *m*)/*k_i_* is the predicted state variable at *k_i_* + *m* with the given current plant information *x*(*k_i_*).

Similarly, given the current plant information *x*(*k_i_*), the future control increment needs the length of control horizon *N_c_* and it is defined as the number of parameters used to capture the future control trajectory. To obtain an optimal control sequence, the defined cost function is minimized in each time step and it can be written according to [15] as follows:

$$J=\sum_{i=1}^{N_{p}}{\left({\hat{y}}_{p}\left(k+i/k\right)-y_{\text{ref}}\left(k+i\right)\right)}^{T}Q({\hat{y}}_{p}\left(k+i/k\right)-y_{\text{ref}}\left(k+i\right))+\sum_{i=1}^{N_{c}-1}u{\left(k+i\right)}^{T}Ru(k+i)$$

where *Q* and *R* are the weighting matrices that tune the relative importance of the output vector's elements as well as the magnitude of the control effort. *y_ref_* is the reference trajectory of vehicle 2. QP formulation is solved for model predictive control problem and adapted for some engineering projects.

## 5. Simulation and Analysis

In this section, comparative analysis of vehicle driving state prediction is studied based on the HISS and a traditional vehicle-mounted sensor system. The desired vehicle distance can be calculated using Equation (9), where, *x_r_*_,0_∈[2,5] m is the minimum vehicle safety distance, *t_hw_*_,_*_d_* = 1.2 s. Therefore, the desired vehicle distance can be calculated at about 15.768 m. According to the above two environment perception methods, we can obtain the reference vehicle distance error is:

(10) $$\Delta {\text{s}}_{1}=\left|16.67-15.768\right|=0.902\;\text{m}$$

Under the veDYNA environment, the reference vehicle distance error is:

(11) $$\Delta {\text{s}}_{2}=\left|16.67-16\right|=0.67\;\text{m}$$

**Figure 10.**
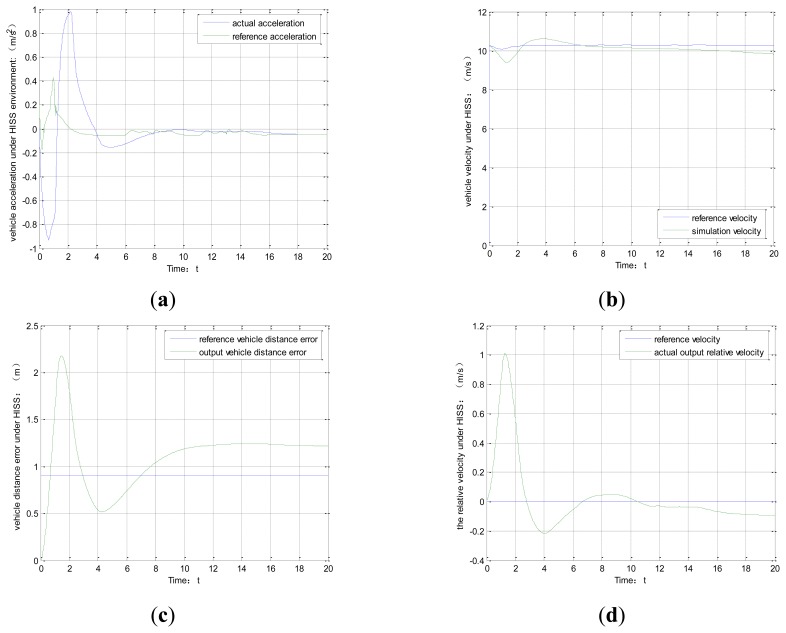
(**a**) vehicle acceleration. (**b**) vehicle velocity. (**c**) vehicle distance error. (**d**) vehicle relative velocity.

Suppose the initial vehicle distance error is 0, at the sampling time *t*_0_, system starts working and model predictive control approach is used to predict vehicle driving state at the next time *t*_1_. The simulation process is shown in Figure 10. Figure 10a and b show the requirement that studies vehicle environment perception under HISS. The acceleration can effectively track the reference acceleration. Figure 10c shows the vehicle distance error between the reference vehicle and the actual distance. According to the state constraints conditions, the distance error lies in the range, which demonstrates that the results are feasibility. Figure 10d shows the relative velocity tracking routine. Overall, the method using HISS to realize the vehicle driving state prediction is accurate.

**Figure 11.**
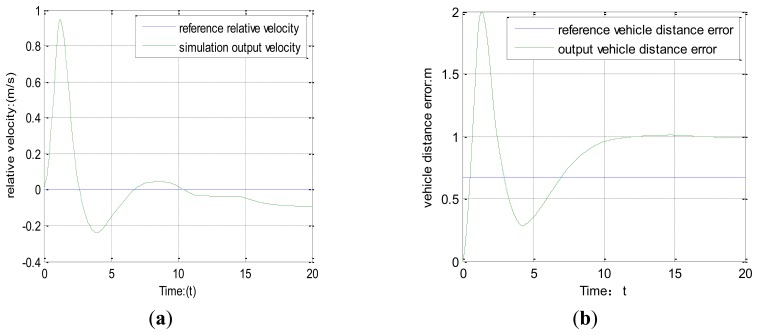
(**a**) The relative vehicle velocity. (**b**) Vehicle distance error.

As to the traditional vehicle-mounted sensor environment perception system, model predictive control is used to predict the vehicle driving state. The simulation process is shown in Figure 11a and b.

Figure 11a shows the relative vehicle velocity under the traditional vehicle-mounted sensor system environment, indicating the simulation results can effectively track the reference velocity. Figure 11b shows the vehicle distance error is in the range of state constraints, which can demonstrate that the system is accurate.

From Figure 10b and Figure 11a, we can reach the conclusion that using the two environment perception systems can effectively track vehicle distance errors. Using a vehicle-mounted sensor system to realize vehicle distance has slightly smaller errors, while using the HISS can keep the vehicle distance error stable. Similarly, the relative vehicle velocity can arrive at the range of state constraints and maintain system stability. The simulations results show that using HISS to realize vehicle driving state prediction in the next period can arrive at the same results as when a vehicle-mounted sensor system is used to realize vehicle driving state prediction. Meanwhile, we can further draw the conclusion that using HISS to realize vehicle environment perception can ensure system stability, thereby demonstrating the method feasibility.

## 6. Conclusions

A Highway Intelligent Space System (HISS) is proposed in this paper to research vehicle driving state in the next time period when a vehicle drives at a high speed. First, the composition, principles and system structure of the HISS are introduced. The vehicle driving state is obtained using the HISS. Second, a traditional vehicle-mounted sensor system is used to study vehicle driving state prediction in the veDYNA environment. Meanwhile, model predictive control is used to research the prediction process. Third, comparative analysis of vehicle driving state prediction between HISS and the traditional vehicle-mounted sensor system is studied in the MATLAB environment. Finally, the simulation results demonstrate that using HISS can realize vehicle driving state prediction and ensure vehicle driving process stability. Meanwhile, the advantages of HISS can provide advance road information far from the vehicles, which provides vehicles with warning information about the road situation ahead.
